# From Akute Primäre Verruckheit to Bouffée Delirante: The background of Acute Transient Psychosis

**DOI:** 10.1192/j.eurpsy.2022.519

**Published:** 2022-09-01

**Authors:** J. Romão, M. Gonçalves, R. André, F. Félix, R. Saraiva, M. Abreu

**Affiliations:** Centro Hospitalar Universitário Lisboa Norte, Psychiatry, Lisboa, Portugal

**Keywords:** acute transient psychosis, atypical psychosis, bouffee delirante, cycloid psychosis

## Abstract

**Introduction:**

Ever since the end of the 19th century that descriptions of acute and transient psychosis (ATP) have been found in the literature. Psychiatrists from different countries gave different names for these types of episodes, throughout the ages. Those early descriptions were an important part of the development of the concept of acute and transient psychotic disorders (F23: ICD-10).

**Objectives:**

This review aims to provide historical background of the development of different concepts to describe ATP.

**Methods:**

Non-systematic review of literature on acute and transient psychotic disorders, bouffee delirante, brief psychotic disorder, atypical psychosis.

**Results:**

In 1876, K.Westphal introduced the term *akute primäre Verruckheit*, refering to a sudden paranoia associated with delusion ideas and hallucinations. In 1895, Magnan described *Bouffée delirante*, characterized by a recorrent, sudden psychosis with polymorphic symptoms. Later (1924), the term cycloid psychosis was introduced by K.Kleist: phasic psychosis with good prognosis. Different concepts appeared throughout history: psychogenic psychosis (Wimmer,1916); atypical psychosis (Mitsuda,1942), holodisfrenia (Barahona,1957). Nowadays, the classification systems include many of these concepts in the same categories: Schizophreniform disorder, Brief psychotic disorder (DSM-5), and ATP (F23 in ICD-10).

**Conclusions:**

All throughout the History of Psychiatry, there was an evolution of concepts associated to ATP. They were strongly influenced by different time epochs. It is important to have context on the historical background of the concepts used in the contemporaneous Psychiatry. Diagnosis is challenging due to their heterogeneous presentation. There are not many studies available, because of ATP’s low diagnostic stability.

**Disclosure:**

No significant relationships.

